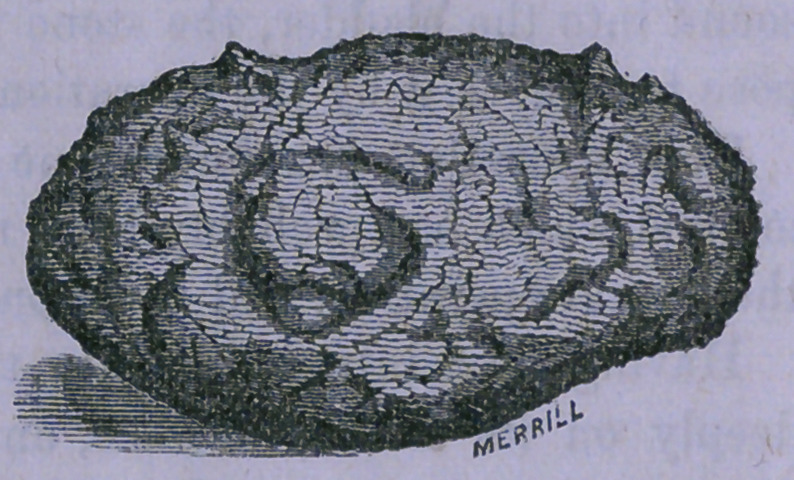# Surgical Clinics by Dr. Edwin Powell

**Published:** 1866-01

**Authors:** R. M. Lackey


### CLINICAL REPORTS.

RUSH MEDICAL COLLEGE DISPENSARY,
January 3, 1866.

### SURGICAL CLINICS BY DR. EDWIN POWELL.

REPORTED BY R. M. LACKEY, M. D.

*Tumor on the Left Side of the Neck—Operation for its Removal—Laceration of the External Carotid Artery—Recovery.*

Female, aged 30 years, married; is anemic and of nervous temperament. Tumor appeared about a year ago, and has gradually increased in size until it has become as large as the fist, and is quite a deformity. The tumor is partly beneath the sterno-mastoid muscle, and is firmly attached to the structures around it. The patient was placed under the influence of chloroform, and the tumor removed: owing to its firm attachments to the vessels, the external carotid artery was lacerated. A ligature was placed on it, however, and one other vessel, and but little blood was lost during the operation. The tumor proved to be cystic, with very thick walls, and containing a small quantity of thick greenish matter.

Jan. 6th. The patient has had a severe cold and cough, which rendered her condition unfavorable for an operation at the time it was done. There has been some swelling about the throat, which has been troublesome; but the wound looks well, and her condition as favorable as could be expected.

Jan. 10th. The swelling has subsided, and the wound looks well and is healing rapidly.

Jan. 17th. Wound nearly healed. Patient leaves to-day for her home in Minnesota.

---

January 6, 1866.

CASE I. *Stone in the Bladder—Lithotomy by the Lateral Method—A large Phosphatic Calculus Removed—Patient doing well.*

The first case we have to present to-day is one of stone in the bladder. This boy is 16 years of age. He is, as you see, rather small and imperfectly developed for one of his age. Says he has had this disease eight years. He has frequent and painful micturition; his urine constantly dribbles from him, and when he urinates, the flow is sometimes suddenly stopped, and a change of position is necessary to start it again. This evidence, however conclusive it may seem, should not be relied upon, but resort should be had to sounding. On passing a sound into the bladder, the stone is distinctly felt, and we propose to remove it by the operation of lithotomy.

This operation has undergone many modifications, but the method which has probably been most frequently resorted to is the lateral operation, and is the one we will perform in this case.

Having first introduced into the bladder a sound, grooved deeply on its convex surface, an incision is made, extending from a point about an inch in front of the anus to the left of the median line, downward and outward, to a point midway between the anus and tuberosity of the ischium. This incision is carried down to the grooved sound in the urethra, then using the sound as a director, with a knife such as I show you, and which is called Sir Astley Cooper's probe-pointed bistoury, the incision is carried down to the prostatic portion of the urethra. I say the incision is to extend down *to* the prostatic portion of the urethra, for I do not regard it as necessary to incise the prostatic portion; for, unless the stone be of an extraordinary large size, this portion of the passage will be large enough to allow of its extraction. At all events, care must be exercised to avoid cutting entirely through the prostate gland, and that portion of the pelvic fascia which is reflected upon the neck of the bladder, for, should this be done, infiltration of urine among the tissues would probably take place.

A difficult part of the operation is in getting into the bladder. The knife may pass too far back, and getting behind the bladder, wound the structures, which should be avoided. Great care is required, then, to keep the beak of the knife firmly in the groove of the staff until the incision is completed. Having carried the incision to the point mentioned, the staff is withdrawn, and the finger introduced into the bladder, and the stone felt for and removed with a lithotomy forceps. There is sometimes another difficult step in the operation—namely, in getting hold of the stone so as to bring it out. The jaws of the forceps should be opened wide enough to embrace the largest-sized stone, and with this precaution, the difficulty in question may be avoided.

The patient was then placed under the influence of chloroform and the operation performed.

A cut of the stone is here given. It measures one inch and seven-eighths in its long diameter, and one inch and an eighth in its short, and weighs 383 grains.

Jan. 10th. Patient has suffered some from prostration following ths operation, but is doing as well ns could be hoped for in a case which has existed so long that the nervous system has become affected, as in this boy.

Jan. 12th. Doing well; wound looks better.

Jan. 15th. Doing well.

---

January 9, 1866.

Case II. *Stone in the Bladder—Lithotomy.*

We have to bring before you to-day another case of stone in the bladder. The patient is a child, three years of age, in whom symptoms of the disease were first noticed last August. He has all the rational signs of the disease which were noticed in the case brought before you at our last clinic; and on passing a sound, the stone, though quite small, can be distinctly felt.

Stone in the bladder is most frequent in childhood and old age, but may occur at any period, and, unfortunately, the difficulties of the operation of lithotomy are greater in the very young and the very old than in middle age. The straining which children always do during the operation, even when under the influence of chloroform profoundly, is extremely annoying. On attempting to pass a sound or the staff into this child's bladder, you see how difficult it is on account of the spasmodic contraction of the sphincters, and, indeed, of the spongy portion of the urethra. But you should not be discouraged by this; the presence of the instrument in the urethra will, after a time, cause the spasmodic action to cease, and you may proceed with the operation.

There are several varieties of urinary calculi—namely, uric or lithic acid, phosphate of lime, phosphate of ammonia and magnesia, oxalate of lime, cystic oxide, and the xanthic acid. These constitute the principal varieties. The lithic acid and phosphate of lime are the most common; the first of these may be known by its being of a hard-brown color, inodorous and smooth. The phosphate of lime is of a pale-brown color, quite smooth, and arranged in lamina.

The patient having been placed under the influence of chloroform, the operation was performed, and a phosphatic calculus removed.

Jan. 15th. Patient doing well; wound looks well.

---

*Fistula in Ano, of eight years' standing—Operation.*

The next case I have to bring before you to-day is one of fistula in ano. This man has had the disease eight years; it has not affected his general health much, and certainly does not in this case exist in conjunction with pulmonary tuberculosis, as some believe it does in many cases. I almost covet this man's muscular development. Fistula in ano may be complete or incomplete. In the complete, the sinus extends from an external opening near the anus to one in the gut—forming a false passage, through which gas and feces may escape. A sinus may extend from an external opening near the anus some distance alongside the gut, without opening into it, constituting incomplete or blind fistula.

The treatment for this disease is to cut through the sphincters and allow the wound to heal by granulation from the bottom. It is useless for you to attempt to cure these cases by stimulating injections, etc., for you cause your patient equally as great suffering as by the operation, and without curing the disease; and the patient may subsequently come to the city to be operated on when you can just as well do the operation.

You first examine the parts to see if the fistula is complete or incomplete, and if there are any small sinuses branching out from the main passage. You then pass a director through the sinus into the gut, and bring out the end at the anus, then with a bistoury, slit up the structures on a director. If any small sinuses exist, they should be slit up also. Introduce a piece of lint between the edges of the wound to prevent immediate union, and allow it to heal by granulation from the bottom.

## Figures and Tables

**Figure f1:**